# Redefining Dermatomyositis: A Description of New Diagnostic Criteria That Differentiate Pure Dermatomyositis From Overlap Myositis With Dermatomyositis Features

**DOI:** 10.1097/MD.0000000000000222

**Published:** 2014-11-02

**Authors:** Yves Troyanov, Ira N Targoff, Marie-Pier Payette, Jean-Pierre Raynauld, Suzanne Chartier, Jean-Richard Goulet, Josiane Bourré-Tessier, Eric Rich, Tamara Grodzicky, Marvin J. Fritzler, France Joyal, Martial Koenig, Jean-Luc Senécal

**Affiliations:** Department of Medicine, Division of Rheumatology, Hôpital du Sacré-Coeur (YT), University of Montreal School of Medicine, Montreal, Quebec, Canada; Department of Medicine, Divisions of Rheumatology (YT, MPP, JPR, JRG, JBT, ER, TG, JLS), Internal Medicine (FJ, MK), and Dermatology (SC), Centre Hospitalier de l’Université de Montréal, University of Montreal School of Medicine, Montreal; Laboratory for Research in Autoimmunity, Research Center, Centre Hospitalier de l’Université de Montréal (JLS, MK), Quebec, Canada; Veterans Affairs Medical Center (INT), University of Oklahoma Health Sciences Center, and Oklahoma Medical Research Foundation, Oklahoma City, Oklahoma, United States; and Mitogen Advanced Diagnostics Laboratory (MJF), Faculty of Medicine, University of Calgary, Calgary, Alberta, Canada.

## Abstract

Dermatomyositis (DM) is a major clinical subset of autoimmune myositis (AIM). The characteristic DM rash (Gottron papules, heliotrope rash) and perifascicular atrophy at skeletal muscle biopsy are regarded as specific features for this diagnosis. However, new concepts are challenging the current definition of DM. A modified Bohan and Peter classification of AIM was proposed in which the core concept was the inclusion of the diagnostic significance of overlap connective tissue disease features. In this clinical classification, a DM rash in association with myositis in the absence of overlap features indicates a diagnosis of pure DM. However, overlap features in association with myositis allow a diagnosis of overlap myositis (OM), irrespective of the presence or absence of the DM rash. Perifascicular atrophy may be present in both pure DM and OM. Recently, the presence of perifascicular atrophy in myositis without a DM rash was proposed as diagnostic of a novel entity, adermatopathic DM. We conducted the present study to evaluate these new concepts to further differentiate pure DM from OM.

Using the modified Bohan and Peter classification, we performed a follow-up study of a longitudinal cohort of 100 consecutive adult French Canadian patients with AIM, including 44 patients with a DM phenotype, defined as a DM rash, and/or DM-type calcinosis, and/or the presence of perifascicular atrophy on muscle biopsy. A detailed evaluation was performed for overlap features, the extent and natural history of the DM rash, adermatopathic DM, DM-specific and overlap autoantibodies by protein A immunoprecipitation on coded serum samples, and associations with cancer and survival.

Two distinct subsets were identified in patients with a DM phenotype: pure DM (n = 24) and OM with DM features, or OMDM (n = 20). In pure DM, the DM rash was a dominant finding. It was the first disease manifestation, was always present at the time of myositis diagnosis, and was associated with a high cutaneous score and chronicity. Concurrent heliotrope rash and Gottron papules (positive predictive value [PPV] 91%), as well as the V-sign and/or shawl sign (PPV 100%), were diagnostic of pure DM. Anti-Mi-2, anti-MJ, and anti-p155 autoantibodies were present in 50% of pure DM patients and were restricted to this subset (PPV 100%). Cancer was present in 21% of pure DM patients. The 15-year survival was excellent (92%).

In contrast, in patients with OMDM, the first manifestation was proximal muscle weakness or other skeletal muscle-related complaints. The DM rash appeared at diagnosis or at follow-up, was associated with a low cutaneous extent score and was transient. Adermatopathic DM, which was absent in pure DM, was highly predictive (PPV 100%) of OMDM. Overlap autoantibodies (including anti-Jo-1, anti-PL-7, anti-PM-Scl, anti-U1RNP, and/or anti-U5-RNP) were found in 70% of OMDM patients. OMDM was not associated with cancer, but the 15-year survival was significantly decreased (65%).

Perifascicular atrophy occurred as commonly in OMDM (n = 6/20, 30%) as in pure DM (n = 4/24, 17%) patients. These 6 OMDM patients had adermatopathic DM at myositis diagnosis, and only 1 of them developed a DM rash at follow-up, emphasizing the lack of specificity of perifascicular atrophy for pure DM.

In conclusion, using the modified Bohan and Peter classification of AIM allowed identification of OMDM, a new clinical subset of OM. Furthermore, identification of OMDM allowed recognition of pure DM as a new entity that was distinct from OMDM or from OM without DM features. However, the absolute specificity of a DM rash and perifascicular muscle atrophy for the diagnosis of pure DM was lost. The distinctive clinical manifestations and autoantibody profiles presented are proposed as diagnostic criteria to differentiate pure DM from OMDM.

## INTRODUCTION

Dermatomyositis (DM) is a major subset in autoimmune myositis (AIM), and both the DM rash and the presence of perifascicular atrophy have been traditionally regarded as specific findings for this diagnosis.^2^ The International Myositis Assessment and Clinical Studies group has recently proposed guidelines for therapeutic clinical trials in the idiopathic inflammatory myopathies.^13^ The authors concluded that polymyositis (juvenile and adult) and DM (juvenile and adult) were the consensus subsets for clinical trials, and agreed that the presence of the DM rash classifies a patient as having DM.^13^

However, 3 new concepts in the classification of AIM have challenged these definitions. First, histopathologic studies of patients with a DM rash have suggested that there are 2 distinct subsets in DM: DM with vascular pathology (or “myovasculopathy”), and immune myopathy with perimysial pathology (IMPP), first described in patients with anti-Jo-1 autoantibodies.^12^ Damage to intermediate-size vessels, capillary loss, membrane attack complex (MAC) deposition on capillaries and mitochondrial abnormalities are only seen in DM with vascular pathology, while perifascicular muscle atrophy may be present in both subsets.^12^

Second, a modified Bohan and Peter classification was proposed to improve the original classification of Bohan and Peter for AIM.^10,19^ The core concept of this purely clinical classification was the attribution of diagnostic significance to the presence of overlap connective tissue disease features. In this classification, a DM rash in association with myositis in the absence of overlap connective tissue disease features allows a diagnostic of pure DM. However, the presence of overlap features in association with myositis indicates a diagnosis of overlap myositis (OM), irrespective of the presence or absence of a DM rash.^19^ Again, perifascicular atrophy may be present in both subsets.

Third, the 119th European Neuromuscular Center international workshop on trial design in Adult Idiopathic Inflammatory Myopathies introduced a rarely described subset of DM: adermatopathic DM or possible DM sine dermatitis.^7^ Therefore, in the absence of a DM rash, the presence of myositis with perifascicular atrophy on muscle biopsy would allow a diagnostic of DM sine dermatitis or adermatopathic DM.^5,7^

The objective of the present study was to further differentiate pure DM from OM. We used a well-documented cohort of French Canadian patients with a DM phenotype, defined as having a DM rash, and/or DM-type calcinosis and/or the presence of perifascicular atrophy on skeletal muscle biopsy. A close evaluation was performed for the extent and natural history of the DM rash, adermatopathic DM, DM-specific autoantibodies, association with cancer and survival. Pure DM emerged as a distinct subset from OM. However, the absolute specificity of a DM rash and perifascicular atrophy for the diagnosis of pure DM was lost.

## PATIENTS AND METHODS

### Patients

The current study is a follow-up study of a cohort of 100 consecutive adult French Canadian patients with AIM followed longitudinally at the Centre Hospitalier de l’Université de Montréal (CHUM), a tertiary care center composed of 3 university hospitals (Notre-Dame, Saint-Luc, and Hôtel-Dieu hospitals), between March 1967 and April 2001.^19^ The original 5 inclusion criteria for this cohort were as follows. First, only French Canadian patients were eligible. Second, they fulfilled Bohan and Peter criteria for definite, probable, or possible polymyositis or DM at any time during follow-up.^2^ Third, patients were 18 years or older at the time of myositis diagnosis (therefore juvenile DM, as defined by Bohan and Peter, was excluded). Fourth, inclusion body myositis, rare forms of AIM, and non-autoimmune myopathies (such as muscular dystrophies) were excluded. Finally, a biobanked serum sample had to be available for study of autoantibodies.

### Data Collection

Data on history, physical findings, and laboratory investigations were obtained by retrospective medical record review using a standardized protocol, as described.^19^ The project was approved by the CHUM Research Ethics Committee. Written consent was obtained from treating physicians to communicate with and examine patients for further data collection. For the present study, chart review was extended until August 2013. The focus of data collection was the description of the DM features of the cohort. Specifically, we recorded the extent and natural history of the DM rash, the presence of adermatopathic DM, the appearance of DM-type calcinosis on follow-up, the presence of perifascicular atrophy on muscle biopsy, the appearance of cancer within 3 years of the diagnosis of myositis and death. Dermatology consultation was routinely performed and was the standard for any description of the DM rash.

### Scoring DM Rashes

To further characterize the extent of the DM rash, a cutaneous score was developed: a maximal DM cutaneous score of 6 at DM diagnosis, and of 7 at follow-up, was determined for each patient. One point was allotted, for a maximum of 6 points, for each of the following dermatologic manifestations of DM: Gottron papules, heliotrope rash, Gottron sign, V-sign, shawl sign and periungual changes felt to be caused either by DM or scleroderma (cuticular hypertrophy, periungual erythema, dilated capillaries visible with the naked eye). An additional point, only at follow-up, was allotted in the presence of any rash attributed to DM extending to the following regions: face, scalp, external ears, arms, abdomen, buttocks, thighs, knees and toes. Duration of the rash was recorded from the date of diagnosis of DM (or from the onset of the DM rash, if an AIM was already diagnosed) to the resolution of the last active DM skin lesions on follow-up.

### Definitions

Definitions were as follows:

1. *DM rash*: presence of Gottron papules and/or a heliotrope rash;
2. *DM-type calcinosis*: calcinosis of subcutaneous tissues and skin overlying skeletal muscle groups, clearly not suspect of systemic sclerosis-type calcinosis;
3. *DM phenotype*: presence of a DM rash and/or DM-type calcinosis and/or perifascicular atrophy on muscle biopsy;
4. *Classical DM*: presence of a DM rash plus either or both proximal muscle weakness and significant serum creatine kinase (CK) elevation (≥ 500 U/L) (adapted from ref. 5); therefore a patient with a DM rash, proximal weakness and normal CK is considered herein as classical DM;
5. *Clinically amyopathic DM (CADM)*: presence of DM rash without muscle involvement (amyopathic DM) or with minimal muscle involvement (hypomyopathic DM)^5^;
6. *Adermatopathic DM*: synonymous with DM sine dermatitis, was defined at a given time point (herein at myositis diagnosis) by myositis without a DM rash, plus either perifascicular atrophy at muscle biopsy, a DM rash at follow-up, or the presence of DM calcinosis;
7. *Cancer-associated myositis*: defined according to the modified Bohan and Peter classification, that is, presence of cancer within 3 years of myositis diagnosis, plus absence of multiple clinical overlap features plus, if cancer was cured, myositis was cured as well.^4^ In the present study, for classification purposes, a distinct subset for cancer-associated myositis was not employed, that is, patients with DM and cancer at follow-up were classified as DM;
8. *Pure DM*: defined herein according to the modified Bohan and Peter classification, that is, pure DM is myositis plus a DM rash in the absence of overlap clinical features^19^;
9. *Overlap connective tissue disease features are as described*^19^: polyarthritis, Raynaud phenomenon, puffy fingers, sclerodactyly, scleroderma proximal to metacarpophalangeal joints, systemic sclerosis-type calcinosis in the fingers, lower esophageal and/or small bowel hypomotility, carbon monoxide lung diffusing capacity (DLCO) <70% of the normal predicted value, interstitial lung disease on chest radiogram and/or CT-scan, discoid lupus, anti-native DNA antibodies plus hypocomplementemia, 4 or more of 11 American College of Rheumatology criteria for systemic lupus erythematosus, and antiphospholipid syndrome;
10. *Overlap myositis (OM)*: defined according to the modified Bohan and Peter classification, that is, myositis with overlap connective tissue disease features, irrespective of the presence of a DM phenotype.^19^ When defined by both the clinical and serologic criteria,^19^ OM is a myositis with either overlap features or overlap autoantibodies, irrespective of the presence of a DM phenotype;
11. *DM-specific autoantibodies*: include autoantibodies to Mi-2, MJ, p155, and SAE autoantigens^16^;
12. *Overlap autoantibodies*: include autoantibodies to Jo-1 and all other synthetases, scleroderma-associated as well as scleroderma-specific autoantibodies,^10,19^ and autoantibodies to nucleoporins.^11^ Anti-signal recognition particle (SRP) autoantibodies were not considered as overlap autoantibodies, but as necrotizing autoimmune myopathy-specific autoantibodies.

### Serum Autoantibodies

Coded serum samples were biobanked at −80°C, and all studies for autoantibodies were done without knowledge of clinical data or diagnosis. Antinuclear autoantibodies and anticentromere autoantibodies were determined by indirect immunofluorescence on HEp-2 cells (Antibodies Inc., Davis, CA), and anti-topoisomerase I by ELISA, as described.^10^ Addressable laser bead immunoassay (ALBIA) allowed detection of autoantibodies to Jo-1, U1RNP, topo, Ro (Ro60 + Ro52), La, and Sm. The assay was performed by 1 of us (MJF) at the Mitogen Advanced Diagnostics Laboratory (http://mitogen.ca), Calgary, Alberta, Canada.^10^ For sera positive for anti-Ro by ALBIA, the specificity for anti-Ro52/TRIM21 and anti-Ro60 was further determined by ELISA using recombinant human Ro52/TRIM21 expressed in Escherichia coli and native Ro60 purified from calf thymus (INOVA Diagnostics Inc., San Diego, CA). ^10^

### Protein A-Assisted Immunoprecipitation

Sera were analyzed by 1 of us (INT) for autoantibodies by protein A-assisted immunoprecipitation, both for nucleic acid analysis (RNA silver stain) and for proteins (metabolically labeled with 35S-methionine), along with double immunodiffusion.^1,17–19^ These immunoassays detect all of the described antisynthetases (Jo-1, PL-7, PL-12, OJ, EJ, KS, Tyr, and Zo), anti-PM-Scl, anti-SumoAE, anti-RNA polymerase III, anti-Th/To, anti-U2RNP, anti-U3RNP, anti-U5RNP, anti-SRP, anti-Mi-2, anti-p155/140, and anti-MJ.

### Pathology

Skeletal muscle biopsy at myositis diagnosis was performed in 87 patients as described.^19^

### Statistical Analysis

Chi-square analysis was performed for frequency comparisons among subsets (using the Fisher 2-tailed exact test, where applicable). Positive (PPV) and negative predictive values (NPV), odds ratios (OR), and likelihood ratios were calculated using InStat and Prism 6.0 softwares (GraphPad Software Inc., San Diego, CA). The Mann–Whitney U test was used for comparison of group means. Kaplan–Meier curves were constructed to estimate survival, and cumulative survival curves were compared using the log-rank statistic (Cox-Mantel), as described.^15^ The effects of age, sex, and AIM subsets on survival were assessed using Cox proportional hazards regression analysis using WinSTAT software (R. Fitch Software, Bad Krozingen, Germany).^15^

## RESULTS

### Comparison of 100 Patients According to DM Rash Status at Myositis Diagnosis

As a first step to characterize better the clinical features and serum autoantibodies in our DM patients, we separated the complete cohort into 2 subsets according to the presence or absence of a DM rash at the time of myositis diagnosis (Table 1). A DM rash was present in 31 patients whereas 69 patients had none. Overlap features in association with a DM rash were documented in 16% (n = 5/31) of the patients. The most common overlap features were lung involvement due to interstitial lung disease (n = 4 patients) and arthritis (n = 3 patients).

**TABLE 1:**
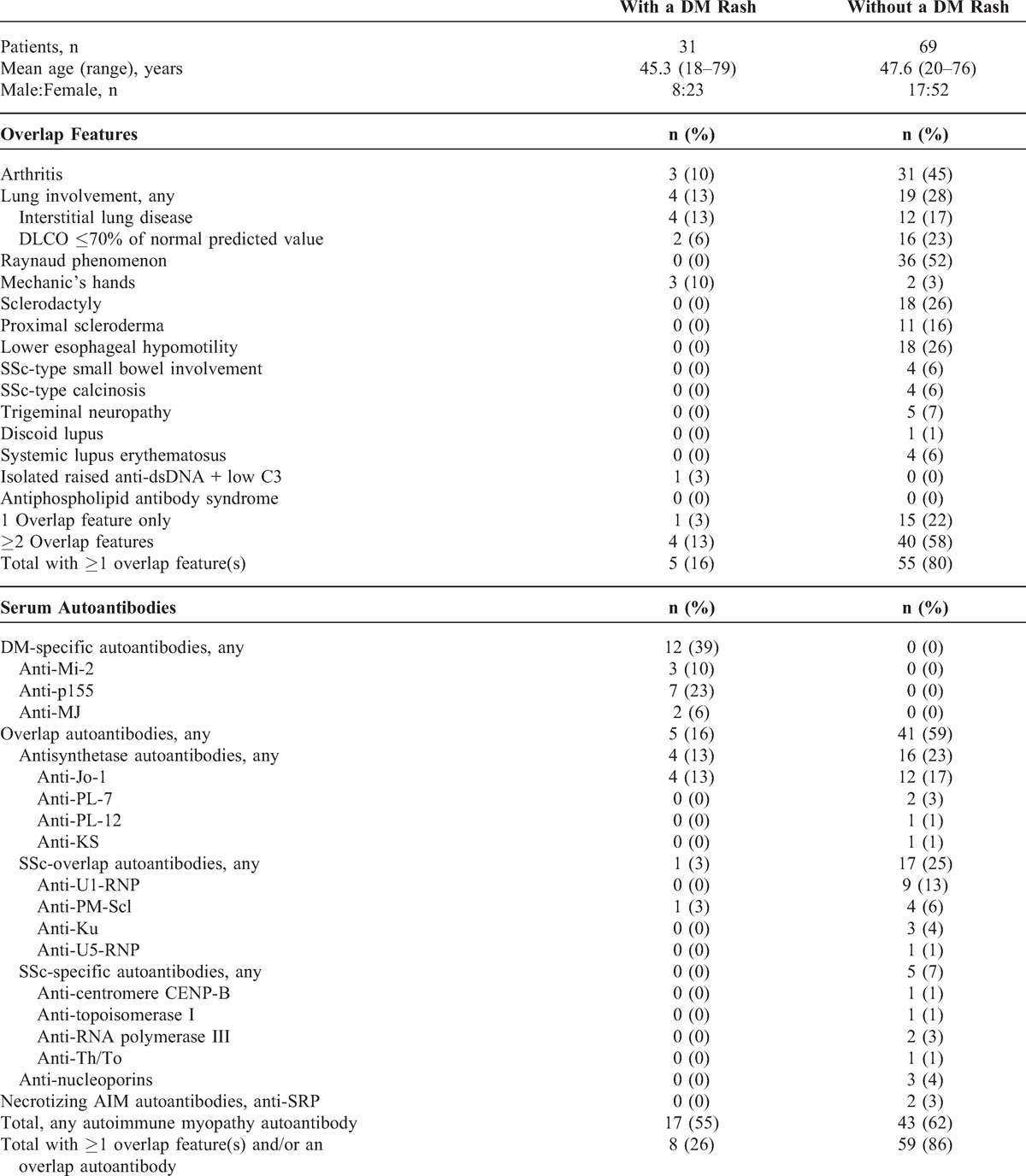
Clinicoserologic Features of 100 Patients With Autoimmune Myositis Classified Clinically by the Presence or Absence of a DM Rash at the Time of Myositis Diagnosis

The DM-specific autoantibodies anti-Mi2, anti-p155, and anti-MJ occurred in 39% (n = 12/31) of patients with a DM rash and were completely restricted to this cohort subset (see Table 1). Overlap autoantibodies anti-Jo-1 and anti-PM-Scl occurred altogether in 16% (n = 5) of patients with a DM rash. In contrast, SSc-specific autoantibodies were completely absent from the DM rash subset. Overall, 55% (n = 17) of patients with a DM rash expressed an AIM autoantibody.

When taken altogether, the data show that a DM rash at diagnosis was associated with overlap features and/or an overlap autoantibody in fully one-quarter of the patients (26%, n = 8/31) (see Table 1).

### DM Rash Is Not Restricted to Pure DM and Occurs as Well in OM

As a second step to refine the characterization of our DM patient population, we further subdivided the complete cohort into 4 subsets according to both their DM rash and overlap status at the time of myositis diagnosis (Table 2). This strictly clinical approach allowed analysis according to the modified Bohan and Peter classification, that is, *pure DM* (defined by myositis plus a DM rash without overlap features), *OM* (myositis plus overlap features with or without a DM rash) and *polymyositis* (myositis with neither overlap features nor DM rash).

**TABLE 2:**
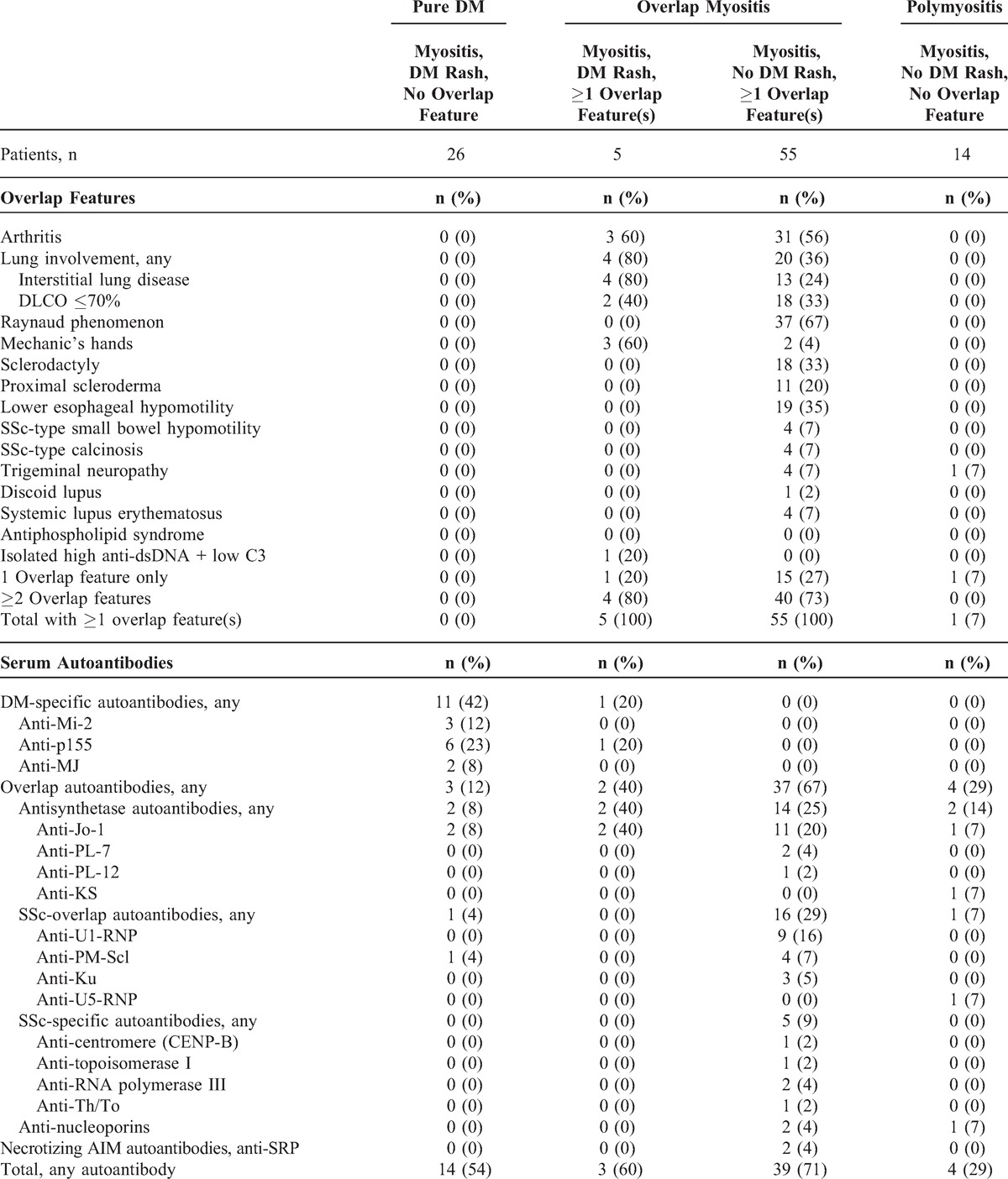
Clinicoserologic Features of 100 Patients Classified Clinically According to Their DM Rash and Overlap Features Status at the Time of Autoimmune Myositis Diagnosis

As shown in Table 2, pure DM was the diagnosis in 26 patients with a DM rash. However, in 5 additional patients with a DM rash, the diagnosis was OM. Of the 60 patients with OM, 5 (8%) had a DM rash. Thus, a DM rash is not absolutely restricted to pure DM and may as well occur in OM.

Antisynthetase autoantibodies were observed in only 8% (n = 2) of patients with pure DM (see Table 2). In contrast, 40% (n = 2) of patients with OM and a DM rash expressed an antisynthetase, compared with 25% (n = 14) of patients with OM without such a rash. We note that anti-Jo1 was the only antisynthetase observed in OM with a DM rash whereas anti-Jo1, anti-PL-7, and anti-PL-12 were all noted in OM without a DM rash. Anti-PM-Scl were noted in a single patient with pure DM. None of the patients with pure DM or OM with a DM rash expressed SSc-specific autoantibodies, anti-nucleoporins, or anti-SRP, whereas all these specificities were observed in OM without a DM rash. Last, antisynthetase autoantibodies tended to cluster with OM, as 80% (n = 16/20) of patients with these antibodies had this diagnosis.

Taken altogether, these results indicate that pure DM emerged as a distinct subset from OM (see Table 2).

### Identification and Frequency of the DM Phenotype

We next took the opportunity of the extensive follow-up of the cohort (mean duration, 15.08 yr; range, 0.33–46 yr), to focus on the 3 criteria defining the DM phenotype, that is, occurrence at any time during disease course of a DM rash and/or DM-type calcinosis and/or perifascicular atrophy at muscle biopsy (Table 3).

**TABLE 3:**
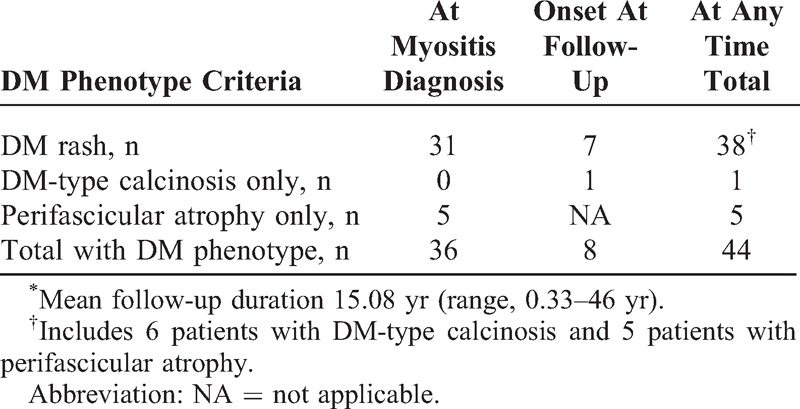
DM Phenotype Manifestations at Any Time in a Cohort of 100 Patients With Autoimmune Myositis^∗^

In addition to the 31 patients who had a DM rash at the time of myositis diagnosis (see Table 2), 7 patients developed a DM rash at follow-up, for a total of 38 (38%) patients with a DM rash at some time during their myositis course (see Table 3). Onset of DM-type calcinosis was noted at follow-up in 7 patients, and it is noteworthy that 1 (14%) of them developed DM-type calcinosis without ever having displayed a DM rash. Perifascicular atrophy was present on muscle biopsy at myositis diagnosis in 10 (10%) patients. However, 6 (60%) of these patients did not have a DM rash at the time of myositis diagnosis, and only 1 of them developed a DM rash on follow-up. Therefore, 5/10 (50%) patients with perifascicular atrophy never displayed a DM rash at any time during their disease course.

Taken altogether, the data indicate that 36 (36%) patients of the cohort had the DM phenotype at the time of myositis diagnosis (see Table 3). At last follow-up, 44 (44%) patients had the DM phenotype, 6 (14%) of whom never displayed a DM rash at any time during their disease course.

### Differentiating Truly Pure DM From OM With DM Features: Analysis of the DM Rash in 44 Patients With the DM Phenotype

Given, as shown above, that a DM phenotype may occur in the absence of any DM rash and that a DM rash is not restricted to pure DM (that is, it may occur as well in OM), further analysis was designed to determine whether differentiating features could be identified among all patients with a DM phenotype to define a truly pure DM disease entity.

As shown in Tables 4 and 5, the 44 patients with a DM phenotype were divided in 2 subsets based on the absence (n = 24) or presence (n = 20) of overlap clinical features, as determined at last follow-up. We then compared the first disease manifestation between these subsets. The DM rash was the first myositis manifestation in 79% of patients without overlap features (n = 19/24) but by contrast in only 10% (n = 2/20) of patients with overlap features (p < 0.0001, OR 34.2, 95% CI 5.9 to 199.2 by the Fisher exact probability test) (Table 4). Myalgia was the first symptom in 17% (n = 4) of patients without overlap features and in 10% (n = 2) of patients with overlap features. Whereas none of 24 patients without overlap features had proximal muscle weakness as the first manifestation, it occurred in 30% (n = 6) of patients with overlap features (p = 0.005, OR 21.9, 95% CI 1.15 to 419.5 by the Fisher exact probability test). Thus, a DM rash as first myositis manifestation strongly predicted a DM phenotype without overlap features, whereas proximal muscle weakness was associated with overlap features.

**TABLE 4:**
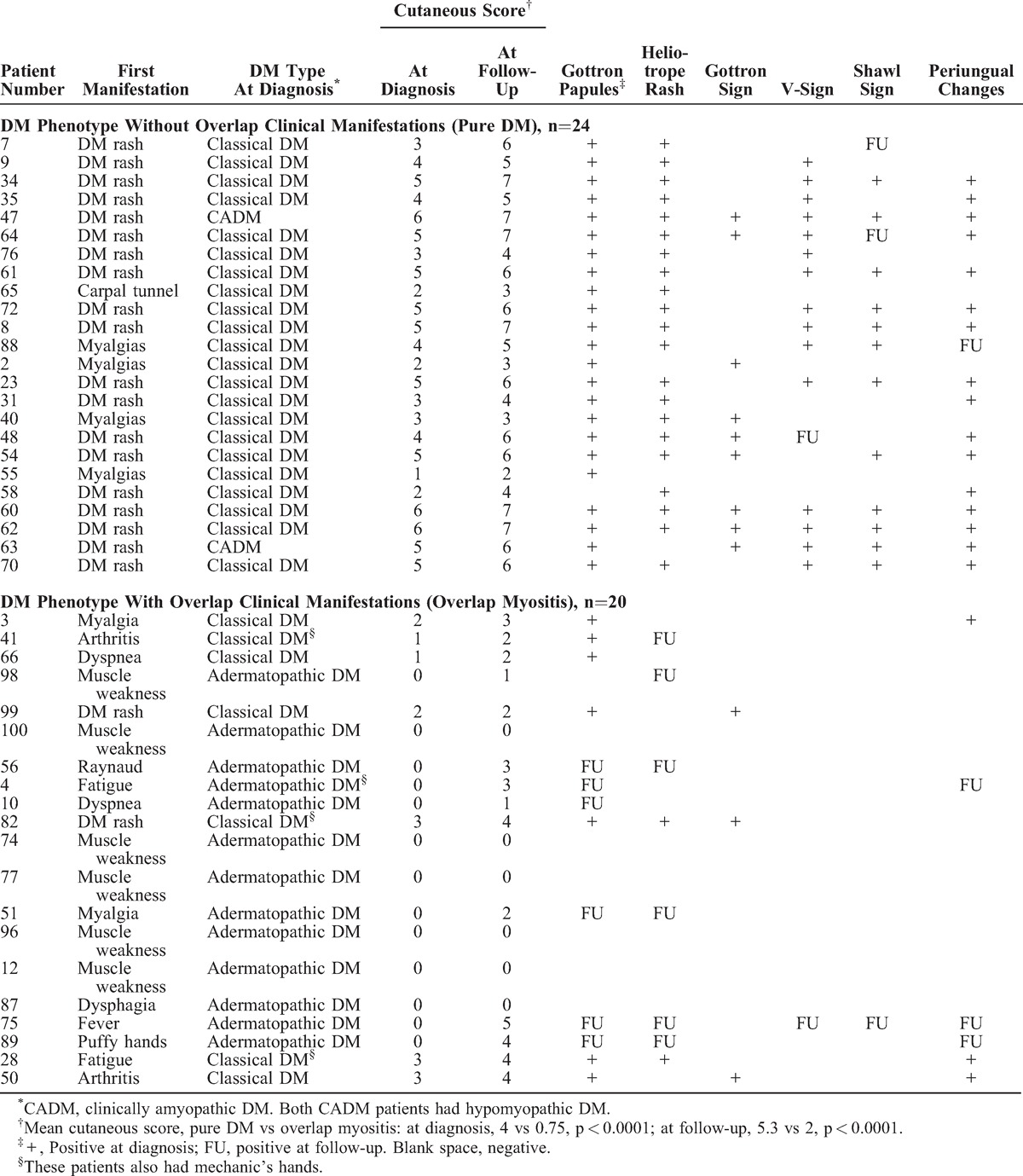
Differentiating Manifestations in 44 Patients With a DM Phenotype, Divided According to the Absence or Presence of Overlap Clinical Features at Last Follow-Up: Cutaneous Manifestations

**TABLE 5:**
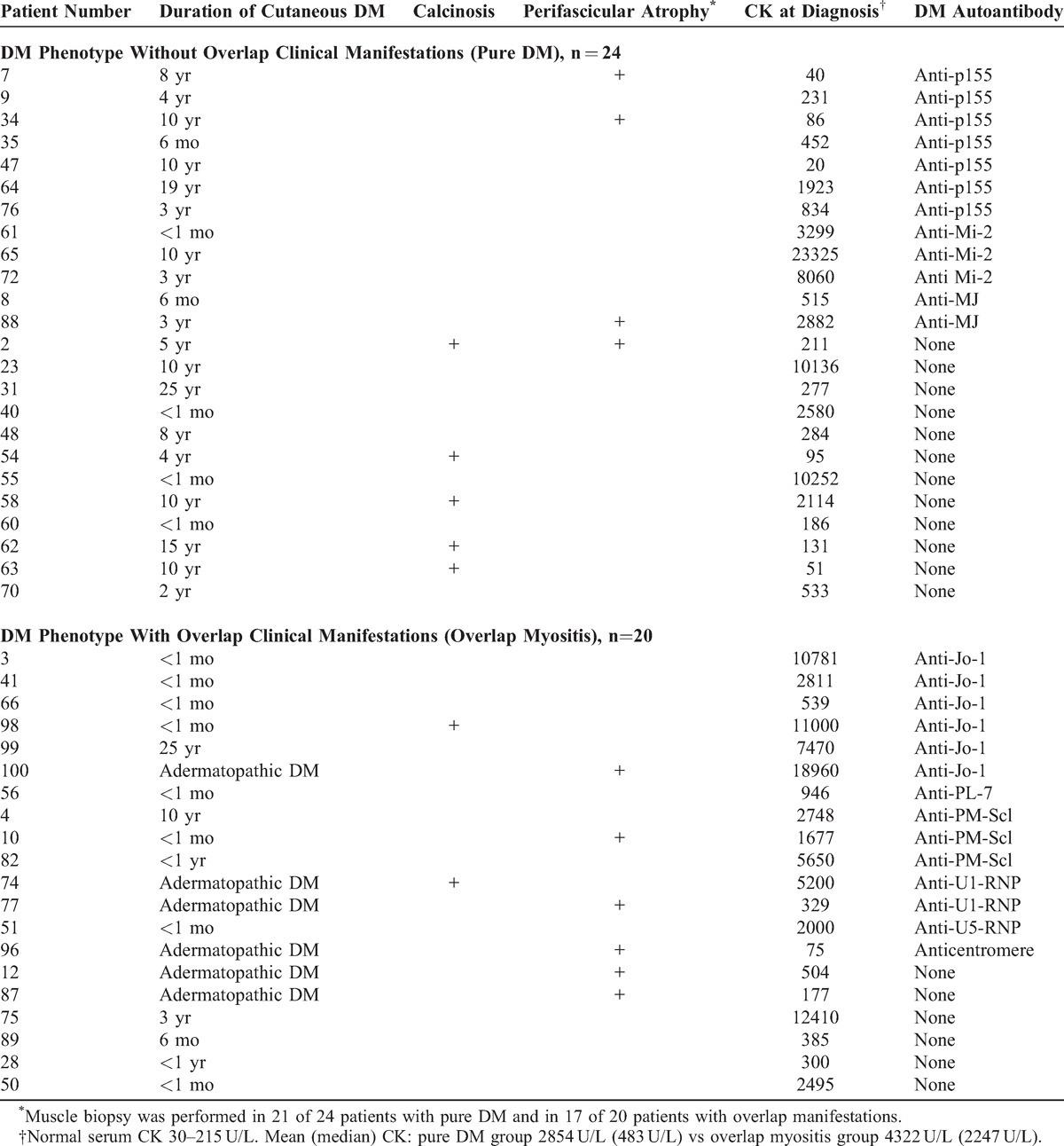
Differentiating Manifestations in 44 Patients With a DM Phenotype, Divided According to the Absence or Presence of Overlap Clinical Features at Last Follow-Up: Duration of Cutaneous DM, Muscle Manifestations, and Serum Autoantibodies

When reexamined at the time of myositis diagnosis, the DM rash was present in 100% (n = 24/24) of patients without overlap features. In this group, a diagnosis of classical DM was made in 92% (n = 22) of patients whereas only 35% (n = 7/20) of patients with overlap features had this diagnosis (p = 0.0001, OR = 20.4, 95% CI 3.7 to 113.5). In the latter group, the most common diagnosis was adermatopathic DM, occurring in 65% (n = 13/20) of patients, contrasting with none of the 24 patients without overlap features (p < 0.0001, OR = 88.2, 95% CI 34.7 to 1667.6) (see Table 4). Among these 13 patients, 8 (61.5%) developed a DM rash and/or DM-type calcinosis at follow-up, and 6 (46%) had perifascicular muscle atrophy.

Second, the cutaneous extent of DM was analyzed and the DM cutaneous score was compared in patients without overlap features to those with such features, as shown in Table 4. In patients classified at follow-up as a DM phenotype without overlap features (n = 24), the mean DM cutaneous score at myositis diagnosis was significantly higher than in those with overlap features (mean score = 4 vs 0.75, 2-tailed p < 0.0001 by Mann–Whitney test, U-statistic = 24.0). Similarly, the DM cutaneous score at last follow-up was significantly greater in the former group than in the latter (mean score 5.3 vs 2, 2-tailed p < 0.0001 by Mann–Whitney test, U-statistic = 40.0). Therefore, both at diagnosis and last follow-up, patients without overlap features, that is, with pure DM, had significantly more extensive DM cutaneous manifestations.

Third, the duration of cutaneous DM manifestations was compared between the 2 groups with a DM phenotype. In the group without overlap features (n = 24), the DM rash was strikingly chronic and recurrent. Thus, 75% (n = 18) of patients without overlap features had rashes lasting more than 1 year compared with only 15% (n = 3/20) of patients with overlap manifestations (p < 0.0001 by the Fisher exact test, OR 17, 95% CI 3.6 to 79). Similarly, 58% (n = 14/24) of the former group of patients still had evidence of active cutaneous DM more than 4 years after myositis diagnosis, whereas only 10% (n = 2/20) of patients with overlap features did so (p = 0.0014, OR 12.6, 95% CI 2.4 to 67). Even 8 years after diagnosis, nearly half (46%, n = 11) of patients without overlap features still had active cutaneous DM (Table 5).

In the group with overlap features, most patients (70%, n = 14/20) had either transient rashes lasting less than 1 month (n = 8) or adermatopathic DM (n = 6) (see Table 5). The 3 patients with overlap features and cutaneous DM persisting beyond 1 year after myositis diagnosis included a patient with anti-Jo-1 autoantibody who had recurrent Gottron papules for more than 25 years and increasingly severe interstitial lung disease; a patient with anti-PM-Scl who had recurrent Gottron papules and periungual changes for 10 years; last, a patient with no known autoantibody who had diffuse cutaneous systemic sclerosis and a persistent DM rash (DM cutaneous score = 5) until his death secondary to scleroderma renal crisis.

### Simultaneous Gottron Papules and Heliotrope Rash at the Time of Myositis Diagnosis Differentiates Truly Pure DM From OM With DM Features

Individual features of the DM rash were also analyzed (see Table 4). In patients classified at follow-up as having a DM phenotype and no overlap features, 96% (n = 23/24) had Gottron papules at myositis diagnosis, 88% (n = 21) had an heliotrope rash, and 100% of patients had at least 1 of these cutaneous signs. In contrast, in patients with a DM phenotype and overlap features, only 35% (n = 7/20) had Gottron papules at myositis diagnosis, only 10% (n = 2) had a heliotrope rash and 35% (n = 7) had either Gottron papules or an heliotrope rash. In patients classified at follow-up as having a DM phenotype and no overlap features, the frequency of simultaneous Gottron papules and heliotrope rash at the time of myositis diagnosis (83%, n = 20) was significantly greater than in patients with a DM phenotype and overlap features (n = 2, 10%) (p < 0.0001, OR 45, 95% CI 7.3 to 276; PPV 91%, NPV 82%, specificity 90%, sensitivity 83%, likelihood ratio for a positive test 8.3).

Thus, in patients with the DM phenotype, the simultaneous presence of Gottron papules and a heliotrope rash at myositis diagnosis was highly suggestive of a diagnosis of pure DM.

### The V-Sign and Shawl Sign Differentiate Truly Pure DM From OM With DM Features

In patients classified at follow-up as having a DM phenotype and no overlap features, 63% (n = 15/24) displayed a V-sign at the time of myositis diagnosis, 50% (n = 12) had a shawl sign and 46% (n = 11) had both signs (see Table 4). Either sign was present in 67% (n = 16) of patients whereas in the group with overlap features, no patient (0%) had a V-sign or shawl sign at myositis diagnosis (p < 0.0001, OR 79.5, 95% CI 4.3 to 1484; PPV 100%, NPV 71%, specificity 100%, sensitivity 67%, likelihood ratio 8.3). Only a single patient (with diffuse systemic sclerosis) developed both a V-sign and a shawl sign at follow-up. Therefore, the presence of either or both the V-sign and the shawl sign at myositis diagnosis was diagnostic of pure DM.

### Periungual Changes at Diagnosis Differentiate Truly Pure DM From OM With DM Features

In patients classified at follow-up as having a DM phenotype and no overlap features, 75% (n = 18/24) displayed periungual changes at the time of myositis diagnosis. In contrast, in patients classified as having a DM phenotype with overlap features, only 15% (n = 3/20) had periungual changes at diagnosis (p < 0.0001, OR 17, 95% CI 3.6 to 79) (see Table 4).

### Adermatopathic DM Is a Subset of OM

In patients with a DM phenotype and overlap manifestations, the most common DM subset at myositis diagnosis was adermatopathic DM, occurring in 65% (n = 13/20) of patients, contrasting with none of the 24 patients without overlapping features (p < 0.0001, OR = 88.2, 95% CI 34.7 to 1667.6; PPV 100%, NPV 77%, specificity 100%, sensitivity 65%) (see Table 4). Among these 13 patients, 11 had 1 or more overlap clinical features at diagnosis, whereas 2 patients developed systemic sclerosis at follow-up.

Classification as adermatopathic DM at the time of myositis diagnosis was warranted by development at follow-up of a DM rash in 7 these 13 patients, of whom 1 also developed DM calcinosis, and 1 had perifascicular atrophy at muscle biopsy. The remaining 6 patients never developed a DM rash: 1 had isolated DM-type calcinosis at follow-up and 5 patients had isolated perifascicular atrophy at muscle biopsy.

Thus, among all patients with a DM phenotype, adermatopathic DM was a major subset of OM.

### Mechanic’s Hands

Mechanic’s hands were restricted to patients with overlap features (n = 3/20, 15%). These patients had either anti-PM-Scl (n = 2) or no identified autoantibody (n = 1) (see Table 4).

### DM-type Calcinosis

Of the 7 patients with the DM phenotype who developed calcinosis at follow-up, 5 patients had pure DM and none had a DM-specific autoantibody, whereas 2 patients with overlap features had overlap autoantibodies (anti-Jo-1 and U1-RNP, respectively) (see Table 5). We note that DM-type calcinosis was not found in any patient with a DM-specific autoantibody (n = 0/12).

### Perifascicular Atrophy Is Not Restricted to Pure DM

Perifascicular atrophy on muscle biopsy was present in 23% (n = 10/44) of patients with a DM phenotype at the time of myositis diagnosis (see Table 5). As expected, perifascicular atrophy was observed in some patients with pure DM (n = 4/24, 17%). However, the majority (60%, n = 6/10) of patients with perifascicular atrophy did not have any DM rash at myositis diagnosis and were in the overlap group (n = 6/20, 30%) (see Tables 4 and 5). These 6 patients were therefore classified as adermatopathic DM. At prolonged follow-up (mean 7.3 years, range 2 to 15 years), only a single patient developed transient Gottron papules whereas the 5 others remained without any cutaneous DM (see Tables 4 and 5). Therefore, perifascicular atrophy was not specific for pure DM, as it occurred without any DM rash, even at prolonged follow-up.

In patients with pure DM, perifascicular atrophy was associated with the DM-specific autoantibodies anti-p155 (n = 2) and anti-MJ (n = 1). However, in patients with the DM phenotype and overlap manifestations, it was associated with various overlap autoantibodies: anti-Jo-1, anti-PM-Scl, anti-U1-RNP, and anti-centromere (n = 1, each).

### Serum CK Level at Myositis Diagnosis

The mean serum CK level at diagnosis was higher in patients with the DM phenotype with overlap features than in those without overlap features but this did not achieve statistical significance (4322 U/L vs 2854 U/L, 2-tailed p = 0.064 by Mann–Whitney test) (see Table 5). In the former group the median CK value was 2247 U/L vs 483 U/L in the latter group. Serum CK level may be more closely related to the autoantibody present. For example, in patients with anti-p155 (n = 7) the mean serum CK level was 512 U/L, whereas in patients with anti-Jo1 (n = 6) it was 8593 U/L, and in those with anti-Mi-2 (n = 3) it was 11,561 U/L.

### DM-Specific Autoantibodies Differentiate Pure DM From OM With DM Features

As seen in Table 5, DM-specific autoantibodies to p155, Mi-2 and MJ autoantigens were present in 50% (n = 12/24) of the patients with a DM phenotype without overlap manifestations but in none (n = 0/20) of patients with overlap manifestations (p = 0.0001, OR 41, 95% CI 2.2 to 755; PPV 100%, NPV 63%, specificity 100%, sensitivity 50%). Thus, the presence of either anti-p155, anti-Mi-2 and anti-MJ was strongly associated with pure DM.

### Patients With Overlap Features, With and Without a DM Phenotype, Share Similar Profiles of Overlap Autoantibodies

To illustrate the similarities between patients classified clinically at last follow-up as OM with a DM phenotype (n = 20) and those classified as OM without this phenotype (n = 46), these 2 patient groups were compared with respect to their profile of autoantibodies, as shown in Table 6. They were also contrasted to patients with pure DM (n = 24). The remaining 10 patients of the cohort with neither a DM phenotype nor overlap features were excluded.

**TABLE 6:**
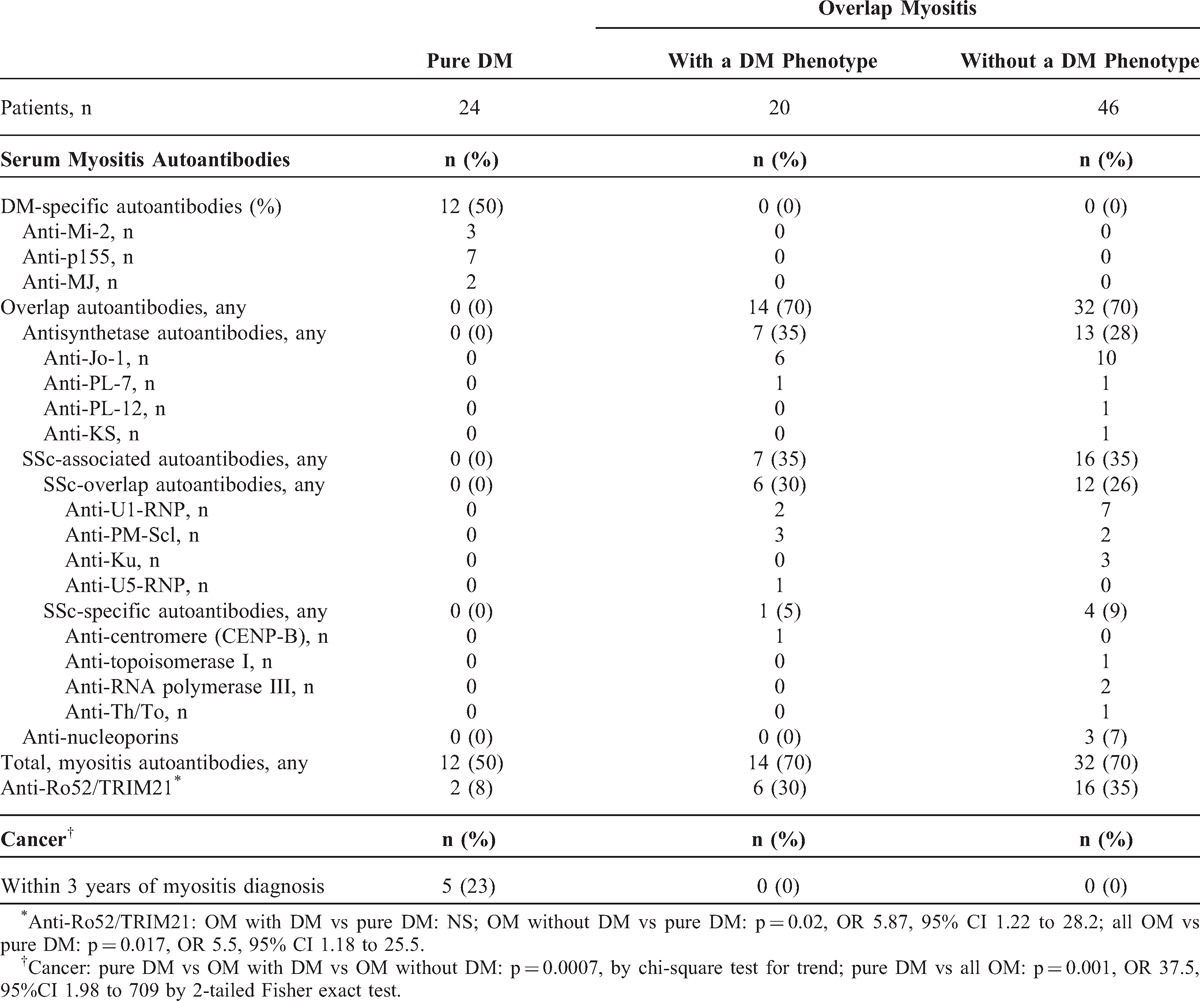
Comparison of Autoantibody Profiles and Cancer in 90 Patients With Either Pure DM or Overlap Myositis With or Without a DM Phenotype as Determined Clinically at Last Follow-Up

The overall frequency of overlap autoantibodies in both groups of patients with overlap features was remarkably similar (70% each) (see Table 6). Specifically, the proportions of antisynthetase autoantibodies (35% vs 28%) and SSc-associated autoantibodies (35% each) were similar. In contrast, the DM-specific autoantibodies anti-Mi-2, anti-p155 and anti-MJ were entirely restricted to pure DM.

### Anti-Ro52/TRIM21 Are More Common in OM Than in Pure DM

The frequency of anti-Ro52/TRIM21 in patients with overlap features with a DM phenotype was greater than in pure DM (30% vs 8%) but this was not statistically significant (see Table 6). However the frequency of anti-Ro52/TRIM21 was significantly greater in OM without DM features than in pure DM (n = 16/46, 35% vs n = 2/24, 8%; p = 0.02, OR 5.87, 95% CI 1.22 to 28.2). When all OM patients were combined, anti-Ro52/TRIM21 were also significantly more common in OM than in pure DM (n = 22/66, 33% vs n = 2/24, 8%) (p = 0.017, OR 5.5, 95% CI 1.18 to 25.5).

### Cancer Is Restricted to Patients With Pure DM

Cancer within 3 years of myositis diagnosis occurred in 5 of 24 (21%) patients with pure DM, and in none of the OM patients with the DM phenotype (n = 20) or without this phenotype (n = 46) (p = 0.0007, by chi-square test for trend) (see Table 6). The frequency of cancer in pure DM (21%) compared with all patients with OM (n = 0/66, 0%) was statistically highly significant (p = 0.001, OR 37.5, 95%CI 1.98 to 709 by 2-tailed Fisher exact test). Thus, patients with pure DM as defined herein were markedly at-risk for cancer within 3 years of myositis diagnosis, but patients with a DM phenotype and OM were not.

Of the 5 patients with cancer, it was detected either at myositis diagnosis (n = 3 patients) or after (n = 2). A single patient had anti-p155 autoantibodies: basal cell carcinoma with persistent myositis was followed by resolution of cutaneous DM and myositis after cancer was resolved. A single patient with anti-Mi-2 had colon adenocarcinoma: colon surgery was followed by persistent remission of myositis, but later cutaneous DM recurred, followed by death several years later secondary to metastatic colon cancer. Breast adenocarcinoma was present in 3 additional patients with pure DM without a DM autoantibody. None of the patients with anti-MJ had cancer.

### Pure DM Is Associated With Better Survival Than OM

**FIGURE 1:**
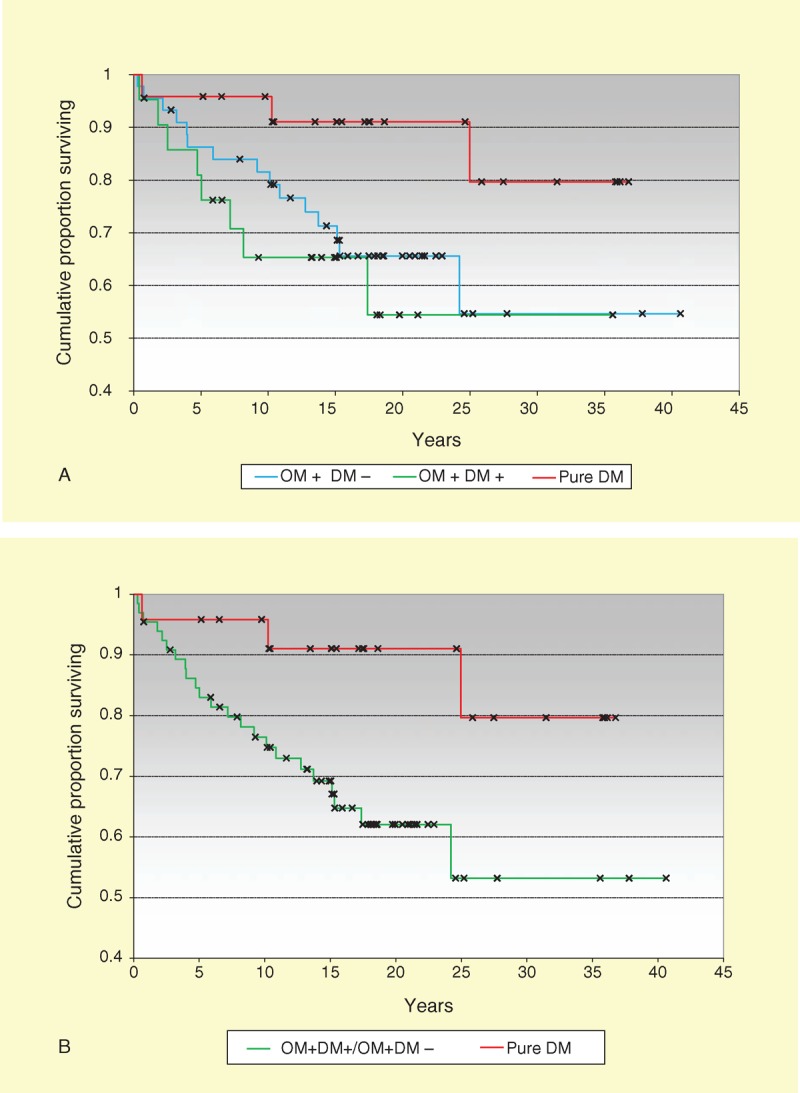
Pure DM is associated with better survival than OM. A. Cumulative 5-year, 10-year, and 15-year survival rates were, respectively, 96%, 92%, and 92% in pure dermatomyositis (pure DM, n = 24 patients), vs 73%, 68%, and 68% in OM with a DM phenotype (OM+DM+, n = 20 patients), vs 87%, 75%, and 65% for OM without a DM phenotype (OM+DM−, n = 46 patients). Survival was significantly different among the 3 subsets (log-rank statistic, p = 0.07). Specifically, survival was significantly better in pure DM in comparison with OM+DM+ (p = 0.04) or OM+DM− (p = 0.03). Survival was not statistically different between the 2 OM groups (p = 0.7). B. When patients from OM+DM+ and OM+DM− groups were combined, survival was even more significantly improved in pure DM (p = 0.02).

As shown in Figure 1A, the cumulative 5-year, 10-year, and 15-year survival rates were, respectively, 96%, 92%, and 92% in pure DM (n = 24 patients), compared with 73%, 68%, and 68% in OM with a DM phenotype (n = 20 patients), compared with 87%, 75%, and 65% for OM without a DM phenotype (n = 46 patients).

Using the log-rank statistic to compare these survival curves, survival was significantly different among the 3 subsets (p = 0.07). Specifically, survival was significantly better in pure DM in comparison with OM with a DM phenotype (p = 0.04) or OM without a DM phenotype (p = 0.03) (see Figure 1A). Moreover, when both OM groups were combined, survival was even more significantly improved in pure DM (p = 0.02) (Figure 1B). Survival was not statistically different between the 2 OM groups (p = 0.7).

When data were further analyzed by Cox proportional hazards regression analysis to take into account potential confounding effects of age at myositis diagnosis and sex, similar results were obtained. Thus, when comparing all 3 groups, myositis subsets accounted for the observed survival irrespective of age at myositis diagnosis and sex (p = 0.063). When pure DM was compared to OM with a DM phenotype, pure DM accounted for a better survival (p = 0.029). Similarly, pure DM accounted for a better survival in comparison to OM without a DM phenotype (p = 0.026). Survival was similar between the 2 OM groups (p = 0.39). Last, patients with pure DM had a significantly better survival than both OM groups combined (p = 0.02).

### Differences Between OMDM and OM Without a DM Phenotype

In order to determine if OMDM patients differed from OM patients without a DM phenotype, both patient groups were specifically compared. Thus, both OM subsets were similar with respect to the frequencies of antisynthetase autoantibodies (35% vs 28%, respectively), anti-Ro52/TRIM21 autoantibodies (30% vs 35%, respectively), and cancer (0% in each subset) (see Table 6). Both subsets also shared similar survival (see Figure 1).

However, OM patients without a DM phenotype differed serologically from those with OMDM. As seen in Table 6, a DM phenotype was absent in patients with anti-Ku, anti-topoisomerase I, anti-RNA polymerase III, anti-Th/To, or anti-nucleoporins, and uncommon in OM patients with anti-U1-RNP autoantibodies. Conversely, the OMDM phenotype tended to cluster in patients with antisynthetase or anti-PM-Scl. However, the small number of patients within each autoantibody-associated subset precluded definitive conclusions.

## DISCUSSION

The presence of a DM rash is an important clinical feature for the diagnosis of DM, and perifascicular atrophy at skeletal muscle biopsy is an important diagnostic feature of DM. However, a true consensus on the definition of DM has not been achieved. The objective of the present study, to better define DM, took advantage of a thoroughly studied cohort of French Canadian patients, including 44 patients with a DM phenotype, defined by the presence of a DM rash, and/or DM-type calcinosis and/or the presence of perifascicular atrophy on muscle biopsy.^19^ All patients were closely monitored for the extent and natural history of cutaneous DM, adermatopathic DM, DM-specific autoantibodies, association with cancer and survival.

These patients were also evaluated for overlap connective tissue disease features. A key methodologic rule in the present study is that pure DM was defined according to the modified Bohan and Peter classification, that is, pure DM is myositis plus a DM rash that were observed in the absence of overlap clinical features.^19^ Therefore, the major differentiating feature between pure DM and OM was whether overlap features were present or not. Using this classification, a new clinical subset of OM was identified, namely *overlap myositis with DM features,* or *OMDM.* Furthermore, identification of OMDM allowed recognition of *pure DM* as a new entity, distinct from OMDM or from OM without DM features.

Table 7 summarizes these findings and proposes diagnostic criteria and distinguishing features, in patients with DM manifestations, that facilitate differentiation of pure DM from OMDM. We submit these criteria for confirmation by other research teams and with the hope that they will be useful to clinicians at first evaluation of patients with suspected myositis.

**TABLE 7:**
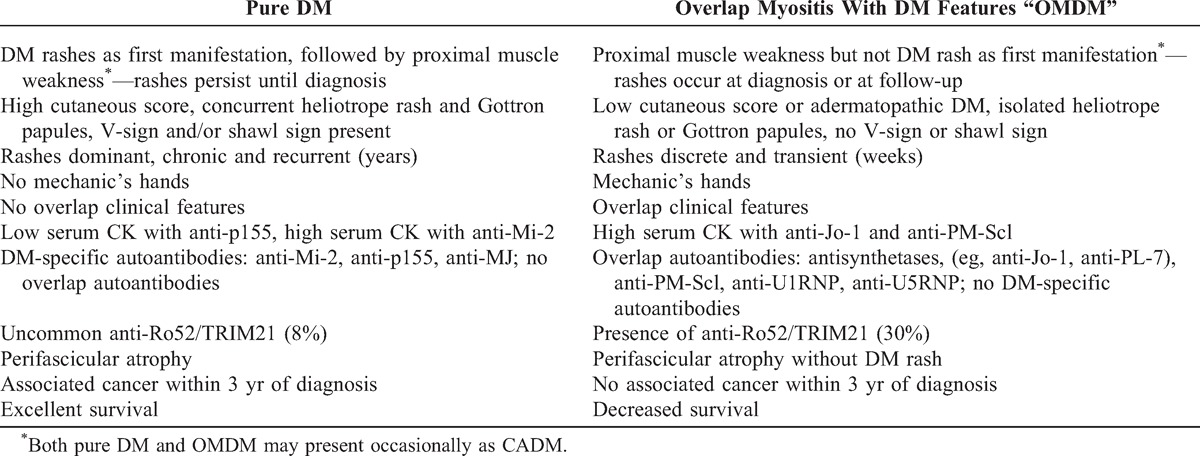
Redefining DM: Proposed New Diagnostic Criteria That Differentiate Pure DM From Overlap Myositis With DM Features (OMDM) at the Time of Myositis Diagnosis

These proposed criteria are elucidated by 5 comments. First, as seen in Table 7, patients with pure DM characteristically display as first AIM manifestation DM rashes that persist until the time of diagnosis. In contrast, in OMDM, DM rashes as first manifestation are rare, occurring typically later in the disease course, either at the time of myositis diagnosis or at follow-up. Proximal muscle weakness is the most common, skeletal muscle-related, first manifestation in OMDM but it is typically absent as first manifestation in pure DM.

Second, as determined by the cutaneous score, DM cutaneous manifestations at diagnosis are much more extensive in pure DM than in OMDM. Moreover, rashes in pure DM are strikingly chronic and may recur for many years. In contrast, most patients with OMDM have transient rashes or adermatopathic DM. In fact, in the present study, adermatopathic DM was strongly predictive of OMDM (PPV 100%).

Concurrent Gottron papules and heliotrope rash at myositis diagnosis is highly predictive of pure DM (PPV 91%) whereas in OMDM these rashes, when present, typically occur singly (see Table 7). Occurrence of the V-sign and/or shawl sign is also highly associated with pure DM (PPV 100%). Interestingly, mechanic’s hands were restricted to OMDM, suggesting that the simultaneous presence of a DM rash and mechanic’s hands may favor a diagnosis of OMDM.

Third, the presence of DM-specific autoantibodies, that is, anti-p155, anti-Mi-2 or anti-MJ, is diagnostic of pure DM (PPV 100%) (see Table 7). In contrast, overlap autoantibodies to various synthetases (for example, anti-Jo-1, anti-PL-7), PM-Scl, anti-U1RNP and U5RNP autoantigens suggest a diagnosis of OM, whether OMDM or simply, in absence of a DM phenotype, of OM.

We note that the overall frequency of overlap autoantibodies in patients with OMDM compared with OM without a DM phenotype was remarkably similar (70%) (see Table 6). Moreover, the frequencies of antisynthetase autoantibodies and systemic sclerosis-associated autoantibodies were closely similar. Only anti-nucleoporin autoantibodies were restricted to patients with OM without a DM phenotype.^11^ The fact that autoantibody profiles in these purely clinically defined patient groups with OM, with or without a DM phenotype, are closely similar and distinct from pure DM, supports the concept of an OMDM subset that is distinct from pure DM.

Anti-Ro52/TRIM21 are significantly more common in OM than in pure DM. Therefore their presence adds diagnostic certainty to a clinical diagnosis of OM (see Table 7). Furthermore, an apparent clinically pure DM associated with anti-Ro52/TRIM21 is more likely an OMDM. We note that anti-Ro52/TRIM21 in patients with systemic sclerosis are also associated with overlap connective tissue disease features.^8^

Fourth, cancer and survival are other features differentiating pure DM from OMDM. Patients with pure DM are markedly at-risk for cancer within 3 years of diagnosis whereas patients with OMDM are at low risk (see Table 7). The observed frequency of cancer in the former group was 21% compared with 0% in the latter. Patients with OM without a DM phenotype had a similar low frequency of cancer (0%) as OMDM patients. With respect to survival, pure DM in the absence of cancer is associated with excellent survival whereas in OMDM survival is markedly and significantly decreased (15-year survival rates: 92% vs 68%, respectively). Taking these cancer and survival data altogether, the overall prognosis is thus markedly different: pure DM is associated with cancer whereas OMDM has a worse survival rate.

Last, perifascicular atrophy at muscle biopsy, an important pathologic clue to the diagnosis of DM,^4^ is indeed observed in pure DM but it is not pathognomonic of this subset, as it is observed as well in OMDM (see Table 7). Supporting the occurrence of perifascicular atrophy in OMDM is its association with various overlap autoantibodies, including anti-Jo-1, anti-PM-Scl, and anti-U1-RNP. Furthermore, of 10 patients with perifascicular atrophy, 50% never displayed any manifestation of cutaneous DM during their disease course. These patients were classified as OMDM with adermatopathic DM. Thus, we propose that, in the absence of DM cutaneous manifestations, the presence of perifascicular atrophy at muscle biopsy of a patient with suspected AIM is suggestive of a diagnosis of OMDM.

A limitation of this study is its clinically defined design, which did not include extensively detailed analysis of muscle biopsy samples for evidence of myovasculopathy and IMPP. Indeed, novel histopathologic classifications of acquired and immune myopathies have recently shed light on the clinical spectrum of AIM.^14^ Six distinct pathologic subsets have been proposed, including myovasculopathy and IMPP that may be associated with a DM rash and perifascicular atrophy. Myovasculopathy is the dominant histologic finding in childhood DM, and also in some adult patients with a DM rash. When compared to IMPP, the distinctive pathologic features of myovasculopathy are mitochondrial changes, MAC deposition on capillaries and damage to capillaries and small to intermediate size vessels.^14^ It is noteworthy that all described patients with this pathologic entity shared 1 clinical feature: the presence of a DM rash. In the original article by Pestronk et al, all 11 patients had a skin rash, muscle weakness, perifascicular atrophy with mitochondrial changes in muscle fibers, and capillary damage with Ulex lectin staining.^12^ In 7 patients, C5b-9 complement deposition in a focal, punctate, capillary-like pattern in the endomysium was observed within areas of perifascicular atrophy. In contrast, the distinctive feature found in patients with IMPP is prominent perimysial connective tissue pathology, with acid phosphatase positive cellularity.^14^ Capillaries are not involved, and C5b-9 complement deposition was rarely seen. Two clinical presentations, with or without a DM rash, correlated with the IMPP pattern on muscle biopsy: patients with anti-Jo-1 autoantibodies, and patients with a myopathy with normal CK but high aldolase.

Thus, it remains to be determined how these histopathologic findings of myovasculopathy and IMPP might segregate with respect to the clinical DM classification proposed herein. We speculate that myovasculopathy would segregate with pure DM, whereas IMPP would cluster with OM.

Although most known AIM autoantibodies were present in our patient population, another limitation of this study is that anti-MDA-5 (originally anti-CADM140) autoantibodies were not assessed. The clinical phenotype of adult American patients with anti-MDA-5 autoantibodies is that of OMDM, that is, a DM rash in association with antisynthetase syndrome features, although antisynthetase autoantibodies are absent.^6^ Anti-Ro52/TRIM21 autoantibodies are common. In addition to myositis, antisynthetase syndrome features noted include symmetric polyarthritis similar to rheumatoid arthritis, interstitial lung disease, mechanic’s hands, and Raynaud phenomenon. Thus, anti-MDA-5-associated clinical phenotype is not that of pure DM, but rather that of OMDM. The clinical spectrum of anti-MDA-5 also includes CADM.^3^ Given that inclusion criteria in our study were focused on myositis and not on clinically amyopathic disease, that is, CADM, this may have resulted in underrepresentation of anti-MDA-5-positive patients. Also, given that in the American cohort of 149 DM patients,^6^ anti-MDA-5 were present in only 7% (n = 11) of patients, our sample size (n = 44 pure DM or OMDM patients) may have been underpowered to allow its detection. Last, ethnogeographic factors influence the frequency of autoantibodies in AIM and other systemic autoimmune diseases.^1,15^ We note that anti-MDA-5 were recently detected in 2 French Canadian patients with the corresponding OMDM phenotype (unpublished observations). These patients were not part of our original French Canadian AIM cohort,^19^ and therefore were not included in the present study.

A large proportion of the AIM patient population reviewed was referred to rheumatology. This patient population is more likely to be referred because of dominant myositis features and to display overlap connective tissue disease features than other patients with DM, such as patients referred to dermatology. For example, in a retrospective chart review of patients presenting with DM to a university tertiary care center (as in the present study), Werth et al reported a clear difference in the presentation of DM to dermatology compared with rheumatology.^9^ Patients presenting to dermatology were much more commonly classified as CADM or hypomyopathic DM than rheumatology patients, whereas the latter were more commonly classified as classical DM. Werth et al concluded that different patients with a DM rash presented to dermatology and rheumatology ^9^. Therefore, in the present study, the proportions of patients classified as pure DM versus OMDM were likely biased by referral to rheumatology.

A corollary to the classification approach employed in the present study is that the absolute specificity of a DM rash and perifascicular atrophy for the diagnosis of pure DM was lost. Although a DM rash is an important clinical feature that suggests the diagnosis of AIM, a true consensus on the definition of DM does not exist. If a DM rash is mandatory for a diagnosis of DM, this study illustrates that the DM rash may not be present at the diagnosis of myositis. Also, if one believes that perifascicular atrophy is a finding pathognomonic of DM, this study illustrates that, paradoxically, some patients with perifascicular atrophy may never develop a DM rash in the course of their disease.

To include patients *without* a DM rash within the definition of DM, new diagnostic criteria will be needed. Indeed, the 119th European Neuromuscular Center international workshop has proposed criteria for the diagnosis of DM in the absence of both a DM rash and perifascicular atrophy.^7^ Muscle biopsy findings of MAC depositions on small blood vessels, or reduced capillary density, or tubuloreticular inclusions on endothelial cells on electronic microscopy, or major histocompatibility antigen type I expression on perifascicular fibers in a patient without a DM rash and perifascicular atrophy on muscle biopsy, would allow a diagnosis of possible DM sine dermatitis.^7^ A cohort of patients with these findings, as well as the findings reported herein, may prove to be very different from classic DM. A broader definition of DM will clearly make DM a more heterogeneous systemic autoimmune disease, with more subsets than recognized thus far.

In summary, the present study identifies 2 novel subsets of AIM in patients with a DM rash and/or perifascicular atrophy: pure DM and OMDM. Striking differences between these subsets were identified. In pure DM, the DM rash was the dominant finding. It was usually the first disease manifestation, was always present at myositis diagnosis and was typically florid and chronic. Anti-Mi-2, anti-MJ, and anti-p155 autoantibodies were present in 50% of pure DM patients. An association with cancer was noted in 21% of patients. Long-term survival was excellent. In contrast, in patients with OMDM the DM rash was rarely the first manifestation of disease, appeared at follow-up and was transient. Adermatopathic DM, which was absent in pure DM, was common in OMDM. In OMDM, autoantibody profiles included anti-Jo-1, anti-PL-7, anti-PM-Scl, anti-U1RNP and anti-U5-RNP similar to OM without a DM phenotype. OMDM was not associated with cancer but survival was markedly decreased.

## ACKNOWLEDGMENTS

The authors thank Gemma Pérez and Haiyan Hou for laboratory assistance and Carole Blouin for secretarial assistance.
